# Wilson’s Disease—Genetic Puzzles with Diagnostic Implications

**DOI:** 10.3390/diagnostics13071287

**Published:** 2023-03-29

**Authors:** Grażyna Gromadzka, Maria Bendykowska, Adam Przybyłkowski

**Affiliations:** 1 Medical Faculty, Collegium Medicum, Cardinal Stefan Wyszyński University in Warsaw, ul. Wóycickiego 1/3, 01-938 Warsaw, Poland; 2 Department of Gastroenterology and Internal Medicine, Medical University of Warsaw, 02-091 Warszawa, Poland

**Keywords:** Wilson’s disease, diagnostics, copper, genetics, epigenetics, genotype, phenotype, ATP7B

## Abstract

(1) Introduction: Wilson’s disease (WND) is an autosomal recessive disorder of copper metabolism. The WND gene is *ATP7B*, located on chromosome 13. WND is characterized by high clinical variability, which causes diagnostic difficulties. (2) Methods: The PubMed, Science Direct, and Wiley Online Library medical databases were reviewed using the following phrases: “Wilson’s disease”, “ATP7B genotype”, “genotype-phenotype”, “epigenetics”, “genetic modifiers”, and their combinations. Publications presenting the results of experimental and clinical studies, as well as review papers, were selected, which concerned: (i) the diversity of genetic strategies and tests used in WND diagnosis; (ii) the difficulties of genetic diagnosis, including uncertainty as to the pathogenicity of variants; (iii) genetic counseling; (iv) phenotypic effects of *ATP7B* variants in patients with WND and in heterozygous carriers (HzcWND); (v) genetic and epigenetics factors modifying the clinical picture of the disease. (3) Results and conclusions: The genetic diagnosis of WND is carried out using a variety of strategies and tests. Due to the large number of known variants in the *ATP7B* gene (>900), the usefulness of genetic tests in routine diagnostics is still relatively small and even analyses performed using the most advanced technologies, including next-generation sequencing, require additional tests, including biochemical evidence of abnormal copper metabolism, to confirm the diagnosis of WND. Pseudodominant inheritance, the presence of three various pathogenic variants in the same patient, genotypes indicating the possibility of segmental uniparental disomy, have been reported. Genotype–phenotype relationships in WND are complex. The *ATP7B* genotype, to some extent, determines the clinical picture of the disease, but other genetic and epigenetic modifiers are also relevant.

## 1. Introduction

Wilson’s disease (WND) (OMIM # 277900) was first described in 1912 by Samuel A.K. Wilson as “progressive hepatolenticular degeneration” [1]. Wilson presented the clinical picture of four patients with unique liver and the brain pathology. Soon after, the hepatic accumulation of copper was postulated, and a Kayser–Fleischer (K-F) ring was recognized as a characteristic sign of WND [1,2].

In the 1930s, it was established that WND is inherited in an autosomal recessive manner [3]. In the 1940s, Cummings confirmed that this disease is related to excessive accumulation of copper in the body [4]. In 1955, it was found that patients with WND had a decreased concentration of ceruloplasmin (CP) in the serum. The lack of CP has been considered the main cause of the disease [5]. In 1993, the *ATP7B* gene located on chromosome 13 (13q14.3) was identified [4,6,7,8,9]. The discovery of pathogenic variants of the *ATP7B* gene in patients with WND has opened a new era of diagnosis and research on this disease.

*ATP7B* encodes ATPase7B (ATP7B) (OMIM*606882), which is abundant in the liver, but also in the brain, kidneys, lungs, intestinal epithelial cells, placenta, and mammary glands [10]. ATPase7B is the copper transporter within cells. The structure of ATPase7B includes a phosphatase domain (A domain), a phosphorylation domain (P domain), a nucleotide binding domain (N domain), and eight copper-binding transmembrane TM domains. Copper transport across the membrane is ATP-dependent [11,12].

Pathogenic variants of the *ATP7B* gene are the reason for the formation of ATPase7B which may have a shortened half-life or incorrect conformation. Such protein may mislocalize within the cell. Mutated ATPase7B is also unable to interact properly with other proteins that are important for proper copper metabolism [13,14,15,16]. As a result, copper is not properly transported in the cell, nor is it effectively excreted from the body [17,18,19,20]. This leads to the accumulation of copper in the body, primarily in the liver and brain, but also in other organs leading to the development of clinical symptoms of WND.

WND is characterized by high clinical variability, which causes diagnostic difficulties. In addition to the history and clinical observations, various methods are used in the diagnosis, including imaging tests, biochemical tests of copper metabolism, and genetic tests. The genetic diagnosis of WND is carried out using a variety of strategies and tests that are increasingly able to identify the genetic changes responsible for WND.

The aim of this manuscript was to present various aspects of genetic diagnosis and genetic counseling in WND, information on the *ATP7B* genotype–phenotype relationship in WND, as well as the importance of other genetic and epigenetic factors for the onset and course of WND.

## 2. Methods

The PubMed, Science Direct, and Wiley Online Library medical databases were reviewed using the following phrases: “Wilson’s disease”, “ATP7B genotype”, “genotype-phenotype”, “epigenetics”, “genetic modifiers”, and their combinations. Publications presenting the results of experimental and clinical studies, as well as review papers, were selected, which concerned: (i) the diversity of genetic strategies and tests used in WND diagnosis; (ii) the difficulties of genetic diagnosis, including uncertainty as to the pathogenicity of variants; (iii) genetic counselling; (iv) phenotypic effects of *ATP7B* variants in patients with WND and in heterozygous carriers (HzcWND); (v) genetic and epigenetics factors modifying the clinical picture of the disease. Older works were cited in order to outline the history and introduce the reader to the presented issues. Recent papers were cited to present the current knowledge on the research problem.

## 3. Results

### 3.1. Genetic Diagnosis of WND

The review of the scientific literature shows that the genetic diagnosis of WND is performed with variety of strategies and tests. The large size of the *ATP7B* gene (80 kb) and the high number of pathogenic variants (around 900 according to the Human Gene Mutation Database (https://www.hgmd.cf.ac.uk/ac/index.php, accessed on 1 January 2023; not all of them are disease-causing), and diversity of variants that may be present both in the coding, as well as in the non-coding sequence, make the genetic testing in the WND is a real challenge.

The genetic testing strategies include (i) a single-gene testing, (ii) multigene panels, (iii) exome sequencing or (iv) full genome sequencing [17].

When clinical and biochemical findings strongly suggest a diagnosis of WND, *ATP7B* sequence analysis is usually performed first. Sequence analysis of the 21 exons and flanking intron sequences is recommended, as most clinical variants are located within these DNA regions. Such analysis can be performed using Sanger sequencing, which allows the detection of deletions, insertions, as well as missense, nonsense, and splice site variants.

If the sequencing method detects only one *ATP7B* variant or no variant, the next step should be gene-targeted deletion or duplication analysis to detect exon or whole gene deletion or duplication.

If these strategies fail to detect the two pathogenic *ATP7B* variants, the promoter sequencing and large deletions or insertions should be investigated with probe amplification by multiplex ligation (MLPA). Such *ATP7B* variants account for approximately 4% of all known mutations of this gene [17]. Regarding promoter testing, analysis is usually limited to the first 500 bp [18]; however, mutations located in more distant regions have also been reported, suggesting the need of analysis of the full promoter sequence [19,20]. Finally, if biallelic mutations in *ATP7B* are not detected in a patient with clinically suspected WND, intron sequence testing should be considered. The characterization of deep intron mutations is a challenge given that *ATP7B* has large introns containing a high content of repetitive elements [21], which makes it impossible to analyse it by Sanger sequencing.

Diagnostic tools based on next generation sequencing (NGS) allow for the examination of the entire *ATP7B* sequence, including exons, introns, UTR regions, and the promoter [19].

It is worth noting that NGS also allows the identification of high copy number variants (CNV)—therefore, it can replace the analysis using MLPA [22].

The multi-gene panel, which includes *ATP7B* and other genes of interest, will most likely identify the genetic basis of WND, limiting the identification of variants of uncertain significance, as well as pathogenic variants in genes that do not explain the clinical phenotype. The genes included in the panel, as well as the diagnostic sensitivity of the tests used for each gene, vary by laboratory and may change over time.

Some laboratories use self-designed panels and/or custom phenotypic-focused genetic analysis that includes clinician-specified genes. Panel studies may be performed using sequence analysis, deletion/duplication analysis, and/or other non-sequencing assays.

Comprehensive genomic testing requires no prior assumptions about which gene is likely to be involved. Exome sequencing or genome sequencing are most commonly used.

Different ethnic groups have different frequencies of specific variants of the *ATP7B* [23,24]. In populations where a small number of pathogenic *ATP7B* variants predominate, it is possible to design relatively simple, inexpensive, and low-time-consuming genetic screening tests based on high-resolution melting (HRM), customized microarrays, or restriction fragment length polymorphism (RFLP) tests [25,26,27,28]. For example, in the Czech Republic, as well as in Poland, the screening for the five or six (respectively) most frequent pathogenic variants in *ATP7B* allows the confirmation of diagnosis in 70–80% of patients [29,30]. Gojova et al. developed a genotyping microarray assay allowing the simultaneous detection of 87 mutations and 17 polymorphisms in the *ATP7B* gene. This assay is based on the arrayed primer extension reaction. According to the authors, the chip can be used as a sensitive, fast, and inexpensive tool, facilitating and accelerating genetic screening for WND [26].

Another approach to genetic testing in WND is to study the sequence of selected *ATP7B* exons—those with the largest number of mutations detected in a given population (so-called mutation hotspots) or those with the most frequent mutations typical for a given population. For example, in Saudi Arabia, in 50% of patients with Wilson’s disease, pathogenic variants were detected in three exons (exons 8, 19 and 21) of the ATP7B gene [31].

The genetic testing may not cover the entire gene and may detect variants of unknown clinical significance. The identification of one or two *ATP7B* variants of uncertain significance does not confirm or exclude the diagnosis, as only the detection of the presence of pathogenic variants in both *ATP7B* alleles by means of genetic tests determines the diagnosis of WND. Due to the large variety of mutations in this gene, the usefulness of genetic tests in routine diagnostics is relatively low, especially considering their high cost.

For this reason, even analyses with the most advanced technologies, including next-generation sequencing, require additional tests, including biochemical evidence of abnormal copper metabolism, to confirm the diagnosis of WND [32].

### 3.2. Genetic Counselling

WND is characterized by a typical Mendelian autosomal recessive pattern of inheritance. Given a carrier rate of one in 90 in the general population, the chance that the patient will have an affected child is 1 in 180 [17]. However, higher carrier rates are seen in some populations due to founder variants.

The probability of giving birth to a child with WND in a situation where the partner’s *ATP7B* gene sequence analysis does not show the presence of pathogenic variants is estimated at 0.5% (the presence of variants in the non-coding sequence of the *ATP7B* gene, which is usually not tested, or the presence of de novo variants cannot be excluded). However, if the partner of the affected person is a carrier of the disease-causing variant in the *ATP7B* (i.e., has the pathogenic variant in one allele), the probability of having an affected child is 50%. Partner testing is of great importance. If the presence of the clinical variant in one allele of the *ATP7B* gene is confirmed in the patient’s partner, soon after delivery of the newborn, genetic tests may be performed; such testing is facilitated because it is focused on specific variants previously diagnosed in parents. Prenatal screening is not recommended, because there is effective pharmacological treatment for WND, so its diagnosis in utero is not an indication for the termination of pregnancy. However, opinions on the use of prenatal testing may differ among physicians and families.

The diagnosis of WND obliges the physician to discuss with the patient how to inform family members who have an increased risk of carrying pathogenic variants in one or both alleles of the *ATP7B* gene. Typically, the patient passes the information on to the family themself. However, there are situations when the patient reuses to inform the family. It is recommended that in such a situation, the physician should try to convince the patient of the importance of informing his/her family [33]. The doctor may assist the patient in providing such information by providing him with all the necessary documentation, or even accompany him in such a conversation. This procedure is approved by the European Parliament [34].

If the proband has been diagnosed by molecular testing, genetic testing of the proband’s parents is recommended to confirm that both parents are heterozygous for the pathogenic *ATP7B* variant. Currently, the method in which only two variants detected in the proband are analyzed is considered insufficient to diagnose or exclude WND in parents [35]. Full sequencing of the *ATP7B* gene in suspected pre-symptomatic or atypical parents is recommended. If a pathogenic variant is detected in only one parent and there are no doubts regarding biological maternity and paternity, it is possible that one of the pathogenic variants identified in the proband occurred as a de novo event or as a de novo postzygotic event in the proband mosaic parent [36].

If both parents are known to be heterozygous for the pathogenic *ATP7B* variant, each affected sibling has a 25% chance of having the disease, a 50% chance of being heterozygous, and a 25% chance of not inheriting either pathogenic variant.

If pathogenic variants in *ATP7B* are identified in the proband, the genetic testing of siblings is facilitated, which focuses on the search for specific gene variants previously detected in the proband. Thanks to this, it is possible to perform tests in a short time and at a low cost. This allows you to quickly confirm or exclude the diagnosis of WND. This is important for people in the preclinical period of the disease, when the results of biochemical tests and clinical observations may be difficult to interpret. The diagnosis of WND facilitated by the results of genetic tests allows for the early initiation of treatment, which can prevent or delay the onset of symptoms [37]. It is worth mentioning that siblings with the same genetic variants may have different clinical presentations [38,39,40]. The range of differences in the clinical picture of WND between siblings may depend, among other things, on the age of diagnosis and start of treatment, reflecting the duration of exposure to copper overload.

### 3.3. Determination of the ATP7B Variants Pathogenicity

A diagnosis of Wilson’s disease can be made in a person who has two pathogenic (or likely pathogenic) variants of *ATP7B* detected by molecular genetic testing. To date, more than 900 variants in this gene have been described. Determination of the pathogenicity of variants is important for WND diagnosis. It is not always easy. Sometimes it is necessary to use different procedures to verify the pathogenicity of the detected *ATP7B* variants.

The first step in pathogenicity assessment is functional characterization of of variant—whether it is a frameshift or a nonsense or a missense variant. In the case of frameshift or nonsense variants, it is usually not necessary to use different pathogenicity assessment techniques, as these variants almost always have a significant phenotypic effect. This effect may depend, to some extent, on the location of the mutation within the gene—it will be more pronounced in the case of variant-affecting codons located closer to the 5’ end than to the 3’ end. The problem with pathogenicity usually concerns missense variants. In the case of these variants, the assessment of pathogenicity is often difficult and requires many analyses.

In 1998–2000, John Forbes and Diane Cox, analyzed the *ATP7B* gene variants using a yeast model. Yeast cells contain the ccc2 gene, which encodes a protein homologous to human ATPase7B. The phenotypic effect of the mutant *ATP7B* variants in strain Saccharomyces cerevisiae (*S. cerevisiae*) was analyzed by deleting the ccc2 gene from yeast cells and cloning the wild-type or mutant human *ATP7B* gene into the yeast genome. The mutated cell lines were cultured on a non-fermenting glycerol medium, which is a source of carbon for yeast. The medium of each cell line containing a given variant of the *ATP7B* gene was enriched with copper sulphate to verify the phenotype (wild-type: normal copper metabolism; or mutated: disturbed metabolism). This effect was measured by the intensity of the cellular respiration, as copper is a cofactor of the respiratory chain proteins. In the publication of the authors, the phenotypic effect of five gene WND variants, p.D765N, p.R778L, p.R778Q, p.G943S, and p.P992L, was analyzed [41].

In 2015, Ozlenen Simsek Papur and Ahmet Koc published another study that described the effect of *ATP7B* gene variants in yeast. Four variants have been characterized: p.T788I (c.2363C>T), p.V1036I (c.1036G>A), and p.R1038G-fsX83 (c.311delC) [42]. The *ATP7B* was cloned into yeast expression vectors and specific variants were made using site-directed mutagenesis. The prepared wild and variant forms of the *ATP7B* gene were introduced into ccc2 gene-deficient yeast cells. The growth rate of the transformants in a metal-limited environment was analyzed to separate the *ATP7B* variants into iron-dependent and iron-independent. All yeast transformants containing a non-wild variant of the *ATP7B* gene presented impaired growth [42].

Chandhok et al. developed a model that allowed studying the effect of treatment with zinc or D-penicillamine on the survival of liver cells with different variants of *ATP7B*. The authors documented that the survival of the p.H1069Q mutant, as well as, to a lesser extent, p.C271*, was improved by D-penicillamine treatment. In this study, the p.R778L mutant showed a good response to zinc treatment [43].

The described techniques for pathogenicity testing are time-consuming and expensive. Therefore, only a few ATP7B variants have been studied in this way so far.

The easiest way to estimate the pathogenicity is to compare the frequency of the variant in the WND population and in healthy people. The detection of a given change (especially in the homozygous form) in healthy individuals suggests that it is not pathogenic.

The pathogenicity of variants can also be verified with the use of genetic databases. The Human Gene Mutation Database at the Institute of Medical Genetics in Cardiff (HGMD) (http://www.hgmd.cf.ac.uk/ac/index.php, accessed on 1 January 2023) is a noteworthy database of gene variants that can be helpful in the process of determining the pathogenicity of variants. Another database of note is the Gencards database created in cooperation with the Weizmann Scientific Institute (an Israeli scientific institute) and the Life Map Sciences company, which deals with innovative solutions and tools used in biomedical analysis (http://www.genecards.org/, accessed on 1 January 2023). The National Center for Biotechnology Information provides access to the database “dbSNP” (https://www.ncbi.nlm.nih.gov/snp/, accessed on 1 January 2023), which contains descriptions of single nucleotide polymorphisms (SNP) and many other changes, such as insertions, deletions, microsatellites, and non-polymorphic changes.

If *ATP7B* variant causes a change in an amino acid to another in the protein sequence, the phenotypic effects of such a change depend, among other things, on the physical and chemical properties of the “wild” amino acid and those inserted into the sequence following a variant. Information on the properties of amino acids can be found in many databases. The tool that allows assessing the properties of amino acids is the so-called Venn diagram where amino acids are grouped according to their properties [44]. The phenotypic effect of a genetics variant in a protein depends on the group from which the amino acid that has been substituted in place of the correct one comes from. Smaller changes can be expected if the replaced amino acids come from the same groups, but what is decisive is what feature of the amino acid is most important for the properties of a given protein—it can be charge, size, hydrophobicity, or shape.

Another tool helpful in determining the pathogenicity of variants is SIFT—Sort Intolerant—from Tolerant, which is an algorithm that allows the prediction of whether replacing a given amino acid with another will affect the phenotypic effect at the protein level [45]. The SIFT method is based on comparing the extent to which the tested amino acid residues coincide with the reference amino acid residues, using PSI-BLAST—Position-Specific Iterative Basic Local Alignment Search Tool, a program created by the National Center of Biotechnological Information (NCBI), based on protein and nucleotide sequences.

Tens of new *ATP7B* variants are identified each year. Although many of them directly affect protein expression, an increasing proportion of variants are thought to affect mRNA splicing. The effect of such a variant may be errors in the removal of introns, as well as in the combining of exons. Changes resulted from creation of new intersection sites and abnormal maturation of transcripts have a direct effect in the formation of altered polypeptide chains [46]. Synonymous, non-synonymous, and nonsense variants can interfere with the formation of existing splice sites, as well as cause the formation of new ones. In order to identify splicing variants in 2006, the Human Splicing Finder (HSF) website was created (http://www.umd.be/HSF/HSF.html, accessed on 1 January 2023). 

Many other bioinformatics tools that may be useful in determining the pathogenicity of mutations are described by Thusberg et al. [47].

According to the current guidelines, the terms “pathogenic variants” and “probably pathogenic variants” are usually regarded as synonyms in the clinical setting, which means that both types of variants are considered diagnostically significant and can be useful in clinical decisions [48].

### 3.4. ATP7B Genotype—WND Phenotype Correlations

Many authors have been interested in explaining whether and to what extent the *ATP7B* genotype may determine the clinical picture of WND. The different types of *ATP7B* variants differ in their predicted effects on changes in protein production and/or function. That is why it seemed possible that the diversity of *ATP7B* gene variants may be the responsible for the high variability of the clinical picture of WND.

In 1972, Cox et al. distinguished three types of WND [49]: two of them: “juvenile” (mostly Western European) and “slavic” (mainly Eastern European Slavic) had normal serum CP concentrations, and the third (“atypical”) may be characterized by decreased serum CP levels. At the time, the WND gene was unknown. Based on current knowledge, we can assume that the three types of WD were probably characterized by a different genetic background of *ATP7B*. The slavic type was probably related to the dominance of the p.H1069Q variant, which is most common among Eastern Europeans and is generally considered to be phenotypically “mild” [50].

Analyzing possible *ATP7B* genotype–phenotype relationships in WND a few authors hypothesized that frameshift or nonsense variants of these genes, associated with the premature formation of one of the termination codons, may be responsible for a more severe course of the disease than substitutional variants, because ATPase7B encoded by a gene with such variants has a significantly altered amino acid sequence compared to the normal protein, may have the wrong length, may not be active, or may not be produced at all. Therefore, such variants are often classified as “severe variants”. The protein encoded by a gene with a substitution may differ by only one amino acid from the normal protein, which may have residual functional activity—therefore, the substitutions are usually classified as phenotypically “light” variants. Several published studies, which analyzed the influence of the type of variant on the clinical picture of the disease, confirmed this hypothesis. For example, in the Polish population, “severe” variants were associated with more pronounced alteration of copper metabolism, as well as with an earlier onset of WND symptoms than substitutions [29]. In Chinese population severe mutations were predictive of neurological worsening in the neurological WND patients that received chelator therapy [51]. Additionally, in the Greek population, WND appeared earlier in patients homozygous for one of the following variants: p.L936X (nonsense variant), p.Q289X, and c.2530delA (frameshift variant) than in patients with a substitution variant in both alleles of the *ATP7B* gene [52]. However, some authors have described both early and late onset of WND symptoms in carriers of “severe” variants. For example, in Icelandic patients, one patient carrying the c.2007del7 variant died of fulminant hepatic failure at the age of 16, and the other patients with the same variant had late-onset WND with neurologic predominance [53]. In a study in a Japanese population, no association of the c.1708-5T-G and c.2871delC variants with the WND phenotype was found [54]. In a study conducted on the Greek population, patients—homozygous carriers of one of the three “heavy” variants (p.L936X, p.Q289X, and c.2530delA)—had an earlier onset of WND symptoms and lower serum CP levels compared to patients with two missense variants [55].

Some authors tried to establish the phenotypic effects of the missense variants of *ATP7B*. In one study, the p.V1106I variant was homozygous in two late-onset patients. The p.Arg778Leu variant was associated with early clinical manifestation with a predominance of hepatic symptoms [56]. Additionally, four other variants (p.Glu110Ter, p.Ser1363Phe, p.Cys1104Phe, and p.Val1262Phe) were associated with early symptom manifestation (ages 9 to 12 years), neurological or hepatic [57]. Some previous studies have not found any association between the type of variant and the clinical picture of WND [58,59,60].

In addition to analyses aimed at determining the relation between the type of variant and the clinical picture of WND, studies were also performed on the phenotypic effects of specific variants in the *ATP7B*. The most frequently studied variant was p.H1069Q, which is common in many European populations, including the Polish population. The results of experimental studies indicated that ATPase7B encoded by DNA with this variant is not localized correctly in the Golgi apparatus, but in the endoplasmic reticulum, and has a five-times shorter half-life compared to the normal protein [61,62]. In studies using yeast cells lacking the gene for Ccc2p (a yeast P-type ATPase equivalent to human ATPase7B) into which either the normal human *ATP7B* gene or the p.H1069Q variant has been introduced, it has been shown that this variant only slightly reduces the ability of human ATP7aseB to complement the function of the yeast protein Ccc2p [63]. This observation became the basis for the hypothesis of a mild clinical course of WND in patients with this variant, especially in the homozygous form. This hypothesis was confirmed by clinical observations concerning patients from European populations, including Polish. It has been shown that the p.H1069Q variant (both in the homo- and heterozygous form) is associated with a milder aberration of copper metabolism, as well as with a later onset of the first clinical symptoms of WND compared to other variants. Among p.H1069Q/p.H1069Q homozygotes, a higher frequency of neurological WND was also observed compared to patients with other variants [64].

In all analyses of the relationship between the *ATP7B* genotype and the clinical picture of WND, variability in the course of the disease was found among patients with the same types of variants. Differences in the clinical picture of WND have been observed between members of the same family and even between monozygotic twins. In one of the published reports, one of the described pairs of identical twins was characterized by a similar clinical picture of WND with liver cirrhosis and portal hypertension, thrombocytopenia, and the presence of a KF ring. However, progressive neuropsychiatric symptoms occurred in only one of the sisters. The second described pair of twins showed a different clinical picture despite the identical genotype and similar biochemical parameters of copper metabolism. One sister (A1) developed jaundice at the age of 5–10 years, and liver function decompensation and neuropsychiatric symptoms at the age of 35; her sister was symptom-free at diagnosis at age 39 [38].

Varied phenotypic pictures have also been described among homozygous carriers of the p.H1069Q variant who were members of a large, multi-generational family [39]. Out of four patients with a genetically confirmed diagnosis of WND, one was found to have a neurological form, two had a hepatic form, and one person was diagnosed in the presymptomatic period. Among the four people who died before the tests (in these people, the genetic defect was not determined, but it is highly probable that these people were also p.H1069Q/p.H1069Q homozygotes), three manifested hepatic symptoms and one manifested neuropsychiatric symptoms. Similar observations were published by Cocos et al. Of 50 screened living members of two large families in Romania, five WND patients and two asymptomatic subjects had the p.H1069Q/p.M769H genotype. The authors concluded that both genetic and environmental factors may determine the WND phenotype [65].

In a study performed in Gran Canaria, the Leu708Pro variant was present in twelve homozygotes, four compound heterozygotes, two with only one variant detected [66]; no relationship between genotype and phenotype has been observed among individual groups. A recent European study of over 1300 patients with hepatic or neurological WND also found no association between ATP7B genotype and phenotype [67].

Genotypic–phenotypic analyses in WND are not easy due to the high genetic and phenotypic diversity, as well as a different course of the diagnostic process, difficulties in analyzing the course of the disease, etc.

### 3.5. Rare Genetic Findings

Although WND is known to be inherited in an autosomal recessive fashion, there have been a few reports of families where cases of WND have passed down the generations, which may indicate dominant inheritance [39,68,69,70]. These observations suggest that the diagnosis of WND should be considered even in individuals whose family history indicates an autosomal dominant type of inheritance.

Another rare genetic observation regarding WND was the description of patients in whom three pathogenic variants of *ATP7B* were detected [71]. These observations have significant diagnostic value and indicate that any two detected *ATP7B* gene variants should be shown to be in trans before the laboratory offers predictive or carrier tests.

The next rarely described genetic curiosity about WND may be the description of patients in whom the *ATP7B* gene sequence analysis indicated the possibility of segmental uniparental disomy due to the inheritance of a copy of a given chromosome from one parent [72]. These observations indicate that the possibility of various atypical genetic mechanisms in the pathogenesis of WND should be taken into account in the process of genetic counseling. For the correct interpretation of the mode of inheritance of WND, a thorough genetic examination of the parents, if possible, may be important.

There are also interesting reports concerning the frequency of *ATP7B* gene variants in the general population that may indicate that the frequency of WND may even be about 20% higher than commonly assumed [70]. In this context, it is worth quoting the opinion of Scheinberg [73], who suggested that the correct diagnosis of WND is made only in about a quarter of all patients. Failure to diagnose the disease is associated with a lack of or too late initiation of treatment [74]. It cannot be ruled out that some patients remain undiagnosed until death [75,76].

However, the authors of the aforementioned report suggest that a possible reason for the discrepancy between observed frequency of *ATP7B* variants and the number of patients diagnosed with WND may be the reduced penetrance of some *ATP7B* variants [77,78].

### 3.6. Phenotype in Heterozygous WND Carriers

The frequency of heterozygous WND carriers (HzcWND) is estimated at 1–2% of the general population [79]. To date, several studies have attempted to determine whether HzcWNDs are at risk for abnormalities in copper metabolism and possibly other sequelae.

Some authors reported decreased CP and/or serum copper concentrations in people who were probably heterozygous carriers of WND (they were parents of patients with WND) [80,81,82,83,84].

However, other authors have noted no differences in the concentration of CP and total copper between probable HzcWND (parents, siblings, or children of patients with WND) and the control group [85]. The weakness of these observations was that they were based on the analysis of small groups of people of different ages in whom genetic tests confirming WND carriership were not performed.

A study of a relatively large number of genetically confirmed HzcWND showed that most of them had copper metabolism parameters within the normal range; however, their serum copper concentration and urinary copper excretion were significantly lower than in controls [86]. The values and parameters of copper metabolism showed a relationship with the *ATP7B* variant; HzcWND carrying the p.H1069Q variant had higher serum CP values than those with the other *ATP7B* gene variants.

### 3.7. Genetic Co-Factors

Due to the high variability of the clinical symptoms of WND, which cannot be fully explained by the *ATP7B* genotype, studies were conducted to identify additional factors modifying the clinical course of WND.

It was shown, for example, that carriers of the *2 VNTR (variable number of tandem repeats) allele of the interleukin-1 receptor antagonist (*IL1RN*) gene favored the earlier onset of WND symptoms, especially in patients with the neuropsychiatric form of the disease [87].

In another study by the same authors, in the male population, homozygosity for the *SOD2* (superoxide dismutase gene) rs4880 T allele predisposes to earlier manifestation of WND symptoms. The homozygous *CAT* (catalase gene) rs1001179 TT genotype was associated with a later onset of hepatic and neuropsychiatric symptoms of WND compared to other genotypes [88].

In patients with the neuropsychiatric form of WND, those who had the *MTHFR* (methylenetetrahydrofolate reductase gene) c.1298C allele manifested clinical symptoms 6 years earlier than patients without this allele; diplotype c.677CC/c.1298AA was associated with a 6-year delay in onset of neuropsychiatric symptoms of WND [89].

One study showed that in a general population of symptomatic patients, the *APOE* (apolipoprotein E gene) genotype associated with the presence of ApoE isoforms: ε2, ε3, and ε4 had no effect on the clinical form or age of onset of WND. However, one study showed that among women, carriers of at least one *APOE* ε4 allele manifested first WND symptoms earlier by approximately 4 years earlier than *APOE* ε3/ε3 homozygotes and this effect was especially evident among p.H1069Q homozygotes [90]. Some studies did not report a relationship between the *APOE* genotype and the clinical phenotype of WND [91,92,93].

There are also reports of other genes that were studied as potential modifiers of the course of WND. These include: *SLC31A2* (coding CTR2) p.S29L, *DMT1* encoding the divalent metal transporter protein 1, *ATP7A* (OMIM 300011) encoding the ATP7A protein, *XIAP* (OMIM 300079) encoding the X-linked apoptosis inhibitor, *ATOX* (antioxidant 1 copper chaperone), or the *PRNP* gene encoding the human prion protein (OMIM *176640) on the clinical picture of WND [94,95,96,97,98,99]. Significant genotypic–phenotypic relationships were found for the studied variants of the *SLC31A2* [94] and *PRNP* [95] genes. The relationship of the remaining genes with the clinical phenotype of WND has not been clearly confirmed.

One study found an association between the rs738409 *PNPLA3* polymorphism and moderate/severe steatosis in WND [100]. This gene encodes a protein that has various functions in lipid metabolism and has been previously described as a risk factor for nonalcoholic fatty liver disease [101] and for liver steatosis in patients with hepatitis B [102].

Kluska et al. in a whole exome sequencing study of 248 patients with WND identified variants of the *ESD* (S-formylglutathione hydrolase) and *INO80* (encoding a subunit of the chromatin remodeling complex) genes as potentially associated with increased and decreased risk of neurological manifestation of WND, respectively. This study identified additional rare variants of the *APOE* and *MBD6* genes as factors associated with a lower risk of early-onset WND [103].

The results of the above studies indirectly indicate the importance of processes related to the inflammatory reaction, oxidative stress, homocysteine metabolism, copper transport and accumulation, recycling of sialic acids, lipid metabolism, and epigenetic mechanisms with the clinical picture of WND.

### 3.8. Epigenetic Modifiers

Since it was not possible to identify genetic factors related to the *ATP7B* gene and other tested genes, which would be significantly responsible for differences in the clinical course of WND and could be useful in predictive analyses regarding the course of the disease, experimental and clinical studies were undertaken to determine the significance of epigenetic processes related to DNA methylation and histone methylation and/or acetylation for the clinical course of WND.

Differences in the methylation of different regions of DNA in liver biopsies from patients with WND were analyzed compared to healthy controls and controls with other liver diseases. These studies detected a specific WND epigenetic signature, including 18 genome-wide regions, as well as thousands of others with lower confidence. Differentially methylated regions of WND-specific DNA included liver-specific enhancers and genes encoding proteins involved in folic acid and lipid metabolism and the acute inflammatory response. A similar epigenetic signature was detected in the analysis of blood samples, in which more than 200 regions typical of WND were detected, including those that distinguished patients with hepatic and neurological symptoms. These preliminary results suggest that blood epigenetic biomarkers may be useful in predicting the clinical course of WND [104].

## 4. Conclusions

The diagnosis of WND is complex. It is carried out using a variety of strategies and tests that are increasingly able to identify the genetic changes responsible for WND. Due to the large variety of variants in the *ATP7B* gene, the usefulness of genetic tests in routine diagnostics is still relatively small and even analyses performed using the most advanced technologies, including next-generation sequencing, require additional tests, including biochemical evidence of abnormal copper metabolism, to confirm the diagnosis of WND. Various unusual genetic findings have been published in recent years, including pseudodominant inheritance, the presence of three various pathogenic variants in the same patient, genotypes indicating the possibility of segmental uniparental disomy due to the inheritance of a copy of a given chromosome from one parent, as well as a relatively high frequency of *ATP7B* gene variants that may indicate that the frequency of WND may be about 20% higher than commonly assumed. Genotype–phenotype correlations in WND are complex. The *ATP7B* genotype, to some extent, determines the clinical picture of the disease, but other genetic and epigenetic modifiers are also relevant.

## Abbreviations

WND	Wilson’s disease
KF ring	Kayser–Fleischer ring
CP	ceruloplasmin
NGS	next-generation sequencing
RFLP	restriction fragments length polymorphism
HRM	high-resolution melting
NCBI	National Center of Biotechnological Information
HSF	Human Splicing Finder
